# Gut Microbial Profile Is Associated With Residential Settings and Not Nutritional Status in Adults in Karnataka, India

**DOI:** 10.3389/fnut.2021.595756

**Published:** 2021-02-23

**Authors:** Ojasvi Mehta, Leeberk Raja Inbaraj, Stuart Astbury, Jane I. Grove, Gift Norman, Guruprasad P. Aithal, Ana M. Valdes, Amrita Vijay

**Affiliations:** ^1^Nottingham Digestive Diseases Center, School of Medicine, University of Nottingham, Nottingham, United Kingdom; ^2^National Institute of Health Research (NIHR) Nottingham Biomedical Research Center, Nottingham University Hospitals National Health Service (NHS) Trust and University of Nottingham, Nottingham, United Kingdom; ^3^Department of Community Health, Bangalore Baptist Hospital, Bangalore, India; ^4^Division of Rheumatology, Orthopedics and Dermatology, School of Medicine, University of Nottingham, Nottingham, United Kingdom

**Keywords:** BMI, gut microbiota, undernourished, adults', rural—urban linkages

## Abstract

Undernutrition is a leading contributor to disease and disability in people of all ages. Several studies have reported significant association between nutritional status and gut microbiome composition but other factors such as demographic settings may also influence the adult microbiome. The relationship between undernourishment and gut microbiome in adults has not been described to date. In this study, we compared the gut microbiome in fecal samples of 48 individuals, from two demographic settings (rural and urban slum) in Karnataka, India using 16S rRNA sequencing. Nutritional status was assessed based on BMI, with a BMI of < 18.5 kg/m^2^ classified as undernourished, and a BMI in the range 18.5–25 kg/m^2^ as nourished. We analyzed 25 individuals from rural settings (12 undernourished and 13 nourished) and 23 individuals from urban slum settings (11 undernourished and 12 nourished). We found no significant difference in overall gut microbial diversity (Shannon and Unweighted UniFrac) between undernourished and nourished individuals in either geographical settings, however, microbial taxa at the phylum level (i.e., Firmicutes and Proteobacteria) and beta diversity (unweighted UniFrac) differed significantly between the rural and urban slum settings. By predicting microbial function from 16S data profiling we found significant differences in metabolic pathways present in the gut microbiota from people residing in different settings; specifically, those related to carbohydrate and lipid metabolism. The weighted sum of the KEGG Orthologs associated with carbohydrate metabolism (Spearman's correlation coefficient, ρ = −0.707, *p* < 0.001), lipid metabolism (Spearman's correlation coefficient, ρ = −0.330, *p* < 0.022) and biosynthesis of secondary metabolites (Spearman's correlation coefficient, ρ = −0.507, *p* < 0.001) were decreased in the urban slum group compared to the rural group. In conclusion, we report that the geographical location of residence is associated with differences in gut microbiome composition in adults. We found no significant differences in microbiome composition between nourished and undernourished adults from urban slum or rural settings in India.

## Introduction

In broad terms, undernourishment is defined as nutritional deficiency of energy, protein, and other nutrients causing measurable adverse effects on body composition, function, and clinical outcomes (1, 2). In children, nutritional assessment is based upon wasting, stunting, eutrophy, and weight. However, in adults, it is based on body weight and thinness. Body Mass Index (BMI) < 18.5 kg/m^2^ is used as the standard indicator and anthropometric index for assessing undernourishment (3). As per WHO reports, undernourishment is a global problem affecting 462 million people worldwide (4). In India, undernutrition is the leading disease burden contributing 17% of the total Disability Adjusted Life Years (DALYs) in all age groups (5).

Undernutrition in adults from Low Middle-Income Countries (LMICs) is multifactorial with causes ranging from inadequate dietary intakes due to food insecurity to the presence of underlying co-morbidities which may impair the normal assimilation and absorption of nutrients, likely compounded by environmental and genetic factors (6). Undernutrition associated with energy-inadequate diets and micronutrient deficiencies constrains metabolic capacity resulting in a range of secondary illnesses (7–10). Rural areas in LMIC's show a higher prevalence of undernutrition in comparison to urban areas based on the nutritional shortfalls.

Although poverty and its association with food insecurity is a major risk factor for undernutrition, the etiology of the condition is far more complex than the simple lack of food. Recently, alterations in the gut microbiome have been recognized as part of this cycle (6, 11). The absorption of dietary nutrients is largely determined by the trillions of microbes present in the human gut referred to as the gut microbiome. The human gut microbiome provides several metabolic functions that are not encoded in the human genome, for instance, fermentation of complex polysaccharides, metabolism of proteins and peptides, biosynthesis of vitamins, absorption of ions, and regulation of several host metabolic pathways (12, 13). These functions facilitate the pre-processing of dietary nutrients and efficient harvest of dietary energy for the host. An aberrant microbiota or a microbiota lacking specific microorganisms could, therefore, result in reduced nutrient absorption secondary to chronic inflammation (14–17). Several studies (18–20) have looked at the gut microbiome profile of undernourished children in India and other LMICs, which have shown an altered or dysbiotic microbiota composition compared to healthy controls. Furthermore, randomized controlled nutritional intervention trials targeted specifically to modulate the composition of the gut microbiome have shown to be an effective strategy in improving the nutritional status of undernourished children (21, 22). Previous studies reported that residential location, rural, and urban based lifestyle and dietary habits determine the gut microbiome structure of healthy adults (23–25). These microbiome changes based on urban and rural locations are intriguing as it encompasses the impact of local environment and food availability.

Based on previous findings of the association between the gut microbiome and undernourishment in children, we hypothesized that in Indian adults the gut microbiome composition will be affected by nutritional status and factors associated with location. In the current study, we have assessed the gut microbiome composition and 16S rRNA based functional analysis of 48 adults from rural and urban slum settings in Bangalore district of Karnataka in India with BMI < 18.5 kg/m^2^ (undernourished) and BMI ranging 18.5–25 kg/m^2^ (nourished). This study being the first of its kind in India provides useful insights into how nourishment and demographic settings are related to the gut microbial profile.

## Methods

### Study Setting

This study was conducted in an urban slum [Devarajeevanahalli (DJ Halli)] and a rural village (Thindlu) in Karnataka. The locations for the study were chosen based on convenience as the community health department has been working in these areas for several years and has a good rapport with the community. The urban and rural settings were chosen based on their distinct and contrasting dietary habits, lifestyle and socioeconomic status (26).

### Urban Slum

DJ Halli is one of the largest government's notified slums in Bangalore, extending over 1.15 km with 420 huts with a “registered” population of 2,463 (27). DJ Halli is served by the Urban Health Center run by the Department of Community Health, Bangalore Baptist Hospital. Contrary to the official statistics, the population was estimated as 50,000 (~11,000 huts) based on community discussions and observation (28). In the urban slum area of DJ Halli, it is reported that 75% of the population falls below poverty line with only one-third of the population having a regular source of income (29). This limits the access to fresh, wholesome produce and comprises predominantly processed foods which are cheaper and therefore affordable.

### Rural Village

According to the 2011 census information, Thindlu has a total population of 786 people. It is located 36 km away from Bangalore city (30). In contrast to the urban slum, families, and individuals in the rural village own pieces of land mainly for agrarian and animal husbandry purposes providing them with a steady source of income and therefore a better socio-economic status compared to the urban slum population. Furthermore, housing in the rural village is built-in with better sanitation and hygiene facilities and ventilation. The population in the rural village normally consume fresh, farm-grown staples, and have a better diet overall.

### Recruitment of Study Participants

The current pilot study recruited 25 participants from the rural location and 23 participants from the urban slum. Both men and women with no comorbid illnesses and within the age range of 20–60 years were included in the study from both study locations. Potential participants were approached through home visits and screened based on the inclusion and exclusion criteria. Individuals with any history of concurrent acute illness; chronic gastrointestinal (GI) disease, chronic constipation, chronic diarrhea, abdominal tuberculosis; autoimmune disease such as multiple sclerosis or connective tissue disorders; atopic disease like moderate to severe asthma, eczema, eosinophilic disorders; cerebrovascular or peripheral vascular disease, previous antibiotics exposure in past 4 weeks or who lacked capacity to give informed consent were excluded from the study. Eligible participants were provided with a stool collection kit. Dietary details as provided in the Supplementary Information were collected using a 24-h recall questionnaire by interview and total caloric intakes along with the composition of carbohydrates, proteins and fats were determined. Participants who were included in the study were permanent residents who lived in their respective dwellings for a minimum of 1 year.

### Criteria Used for Assessing Undernutrition Status

BMI was used as the criterion for assessing undernutrition status in the current study according to the standard protocol used to assess nutritional status in India (3). BMI in Indian population is categorized as underweight (BMI < 18.5 kg/m^2^), normal (BMI = 18.5–22.9 kg/m^2^), overweight (BMI = 23.0–24.9 kg/m^2^) and obese (BMI ≥ 25 kg/m^2^) (31). BMI was calculated from the height and weight of the participants. Any male and female in the age group of 20–60 years with BMI range of 18.5 to 25 kg/m^2^ were classified as nourished and BMI < 18.5 kg/m^2^ were classified as undernourished in both rural and urban settings. In the rural area, 12/25 individuals were classified as undernourished and 13/25 were as nourished. In the urban area, 11/23 individuals were classified as undernourished, and 12/23 as nourished.

### Sample Collection, Processing, and DNA Extraction

Fresh fecal samples provided by the participants were received at the collection point and then were transported on dry ice on the same day of collection to Bangalore Baptist Hospital laboratory and stored at −80°C. Frozen fecal samples were then transported to the Wellcome laboratory in Christian Medical College, Vellore for further analysis. DNA was extracted using the QIAamp Fast DNA Stool Mini Kit (Qiagen, Germany).

### Sequencing Data Analysis and Diversity Metrics

The V3–V5 region of 16S rRNA was amplified and processed following the library preparation protocols for MiSeq Illumina platforms (32). The samples were sequenced to 15 million reads with custom barcoding according to the Fadrosh protocol (33). Raw reads were demultiplexed, filtered, and denoized to derive amplicon sequence variants (ASV's) using DADA2 implemented in QIIME2 version 2019.10 (34). Taxonomy was assigned using a pre-trained classifier based on the SILVA Database (35, 36). The abundance of microbes at genus level was used for downstream analysis. Microbial diversities were calculated taking the average of the feature table rarefied to 37,318 reads per sample with 50 iterations.

For alpha diversity estimations Shannon index (37, 38) was calculated which accounts for both, the abundance and the evenness of the species present. It is the sum of the proportion of species *i*, relative to the total number of species (*Pi*) multiplied by the natural logarithm of this proportion (ln(*Pi*)) multiplied by −1. It is represented as *H*′ = −∑*Pi* ln(*Pi*), where *Pi* is the proportion of individuals belonging to species *i*. Beta diversity was calculated by comparing the unweighted UniFrac distances (39) which is a phylogenetic diversity metrics and takes the distance between the unique branch lengths into account. The phylogenetic tree was generated by aligning the representative sequences by multiple sequence alignment using FastTree (40). UniFrac distance between two samples is calculated as the ratio of the unique branch length and the observed branch length. It is represented as UAB = unique/observed where A and B are the two samples; unique is the branch length that leads to OTU's observed in samples A or B, observed is the branch length leading to OTU's either in sample A or sample B.

### Functional Profiling From 16S Data

Significant changes in the functioning of the gut microbiome in relation to the change in the diversity across the study groups were predicted from the16S rRNA data using the marker-gene based functional profiling analytical tool “Tax4fun” available at the MicrobiomeAnalyst Pipeline (41) to transform the SILVA-based ASV's into a taxonomic profile of KEGG (Kyoto Encyclopedia of Genes and Genomics) organisms normalized by the 16S rRNA copy number from National Center for Biotechnology Information (NCBI) annotations. These abundance matrices were then linearly combined with the already obtained (42, 43) functional abundances of KEGG organisms' functional profiles. The KEGG orthologs (KO) were then computed based on the phylogenetic differences between the microbial species. KEGG orthologs were assigned to KEGG pathways based on the automated annotation through the KEGG mapping tool. Since KO can regulate various categories of pathways, we have narrowed them down to higher functional KEGG metabolic pathways.

### Statistical Analysis

Differential microbial abundance across the study groups tested by analysis of the composition of the microbiome (ANCOM 2.0) (44) adjusted for age and gender at 0.9 detection, meaning that there was a significant change in the ASV compared to the rest of the ASVs in the community in at least 90% of the comparisons with an FDR corrected *p*-value of 0.05. Multiple independent *t*-tests were performed to determine the statistical differences between microbial diversities, ANCOM based differentially abundant microbial taxa and functional pathways based on the nutritional status and geographical locations. Spearman correlations were used to determine the association of gut microbial taxa with total calories, estimated nutritional intakes and KEGG functional pathways adjusting for multiple testing (FDR < 0.1). All statistical analyses were carried out in R v3.5.2 and SPSS Version 26.

## Results

### Subject and Clinical Characteristics

A total of 48 patients were recruited for the study with a BMI of 18.5–25 kg/m^2^ classified as nourished and BMI < 18.5 kg/m^2^ classified as undernourished. The details and clinical characteristics are present in Table 1A and Supplementary Table 1A. There were no significant differences in age and gender, based on nutritional status (undernourished vs. nourished) and location (urban slum vs. rural). There were significant differences (*p* < 0.001) in the average caloric intakes and estimated intakes of carbohydrates and proteins between undernourished and nourished individuals from both urban slum and rural settings. Based on the 24 h dietary recalls, the estimated daily intake of calories was less than the recommended daily intake ranging from 1,700 to 2,200 kcal (45). However, the intakes for carbohydrates, protein, and fats followed the recommended daily intakes of carbohydrates (200–320 g), proteins (42–59 g), and fats (25–30 g) in all groups (46) (Table 1A). There was no significant difference in the average caloric intakes and estimated intakes of carbohydrates, proteins and fats based on location (i.e., rural vs. urban slum) as shown in Supplementary Table 1A.

**Table 1:** Characteristics of the study participants based on nutritional status (normal vs. undernourished) or location (rural vs. urban slum).

**Sample-category**	**Rural-nourished**	**Rural-undernourished**	***p*-values**	**Urban slum nourished**	**Urban slum undernourished**	***p*-values**
**(A) Clinical Characteristics**						
*N*	13	12		12	11	
Age in years: mean (SD)	31 (7.01)	35.33 (10.87)	0.244	28.67 (8.78)	31.00 (12.51)	0.608
Gender-Male	6 (46.16)	6 (50)	0.855	6 (50)	5 (45.45)	0.837
Female: *N* (%)	7 (53.84)	6 (50)		6 (50)	6 (54.55)	
Weight in Kg: mean (SD)	57.71 (9.63)	45.27 (5.07)	0.001^**^	58.42 (8.68)	41.94 (5.04)	<0.001^**^
Height in Cm mean (SD)	165.00 (12.67)	194.33 (112.97)	0.361	162.58 (7.28)	158.18 (10.30)	0.247
BMI in kg/m^2^: mean (SD)	21.37 (1.64)	17.45 (0.83)	<0.001^**^	22.05 (2.20)	16.74 (1.14)	<0.001^**^
Caloric Intake per day: mean (SD)	1,656.53 (355.22)	859.66 (85.70)	<0.001^**^	1,901.66 (517.22)	1,150.54 (294.62)	<0.001^**^
Carbohydrates Intake	293.61 (45.18)	209.16 (34.39)	<0.001^**^	313.50 (67.18)	202.09 (10.24)	<0.001^**^
Protein Intake	51.30 (11.45)	42.08 (7.12)	0.025^*^	52.50 (11.55)	39.18 (4.60)	0.002^*^
Fat Intake	31.83 (3.26)	29.83 (3.68)	0.164	31.08 (3.62)	28.09 (3.50)	0.058
**(B) Bacterial phylum relative abundance**						
Actinobacteria mean (SD)	0.059 (0.032)	0.041 (0.032)	0.181	0.045 (0.047)	0.078 (0.046)	0.110
Bacteroidete mean (SD)	0.144 (0.082)	0.172 (0.082)	0.413	0.225 (0.122)	0.165 (0.094)	0.201
Firmicutes mean (SD)	0.744 (0.120)	0.746 (0.078)	0.957	0.609 (0.120)	0.605 (0.113)	0.945
Proteobacteria mean (SD)	0.040 (0.052)	0.033 (0.034)	0.703	0.111 (0.098)	0.139 (0.152)	0.610
Others mean (SD)	0.013 (0.026)	0.008 (0.014)	0.539	0.008 (0.013)	0.011 (0.018)	0.561

*(A) Clinical Traits, (B) Bacterial phylum abundances expressed as relative abundance. The data is expressed as Mean (Standard Deviation). p-values are for independent sample t-tests*.

** p-values significant at the level of ≤ 0.001;

* *p-values significant at the level of < 0.05*.

### Overall Difference and Diversity Indices in the Microbial Community

**Figure 1:**
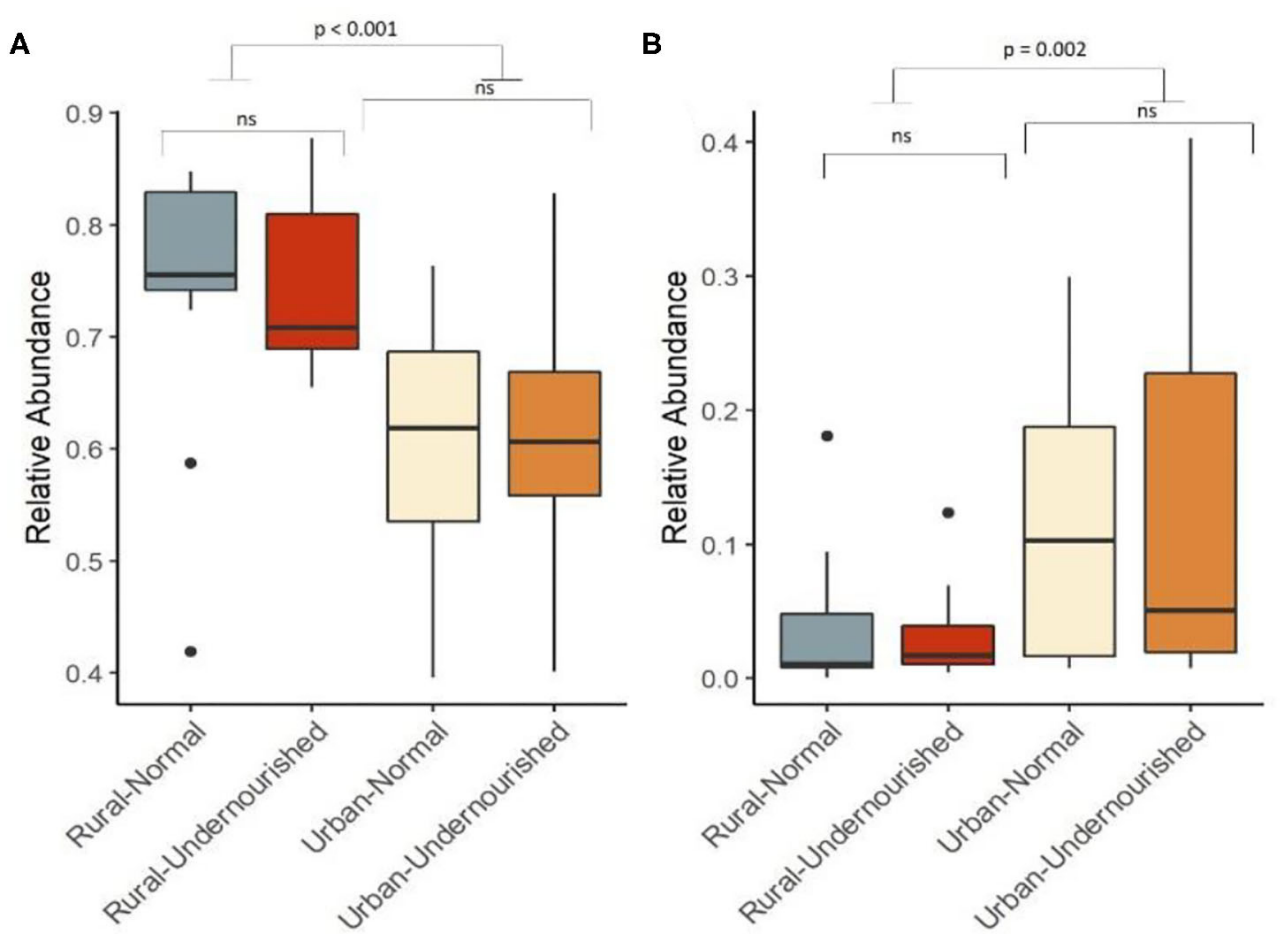
Box and Whisker plot depicting relative abundance of significant phylum. **(A)** Firmicutes and **(B)** Proteobacteria in undernourished and nourished from rural and urban-slum settings. *p*-values indicate statistical significance from independent *t*-tests comparing nourished and undernourished study groups in both the locations (i.e., rural normal vs. rural undernourished and urban normal vs. urban-undernourished) and independent *t*-test based on location (i.e., rural vs. urban-slum). Box-and-whisker plots show high, low, and median values, with lower and upper edges of each box denoting first and third quartiles, respectively. ns indicates non-significant *p*-values. Black dots represent the outliers.

**Figure 2:**
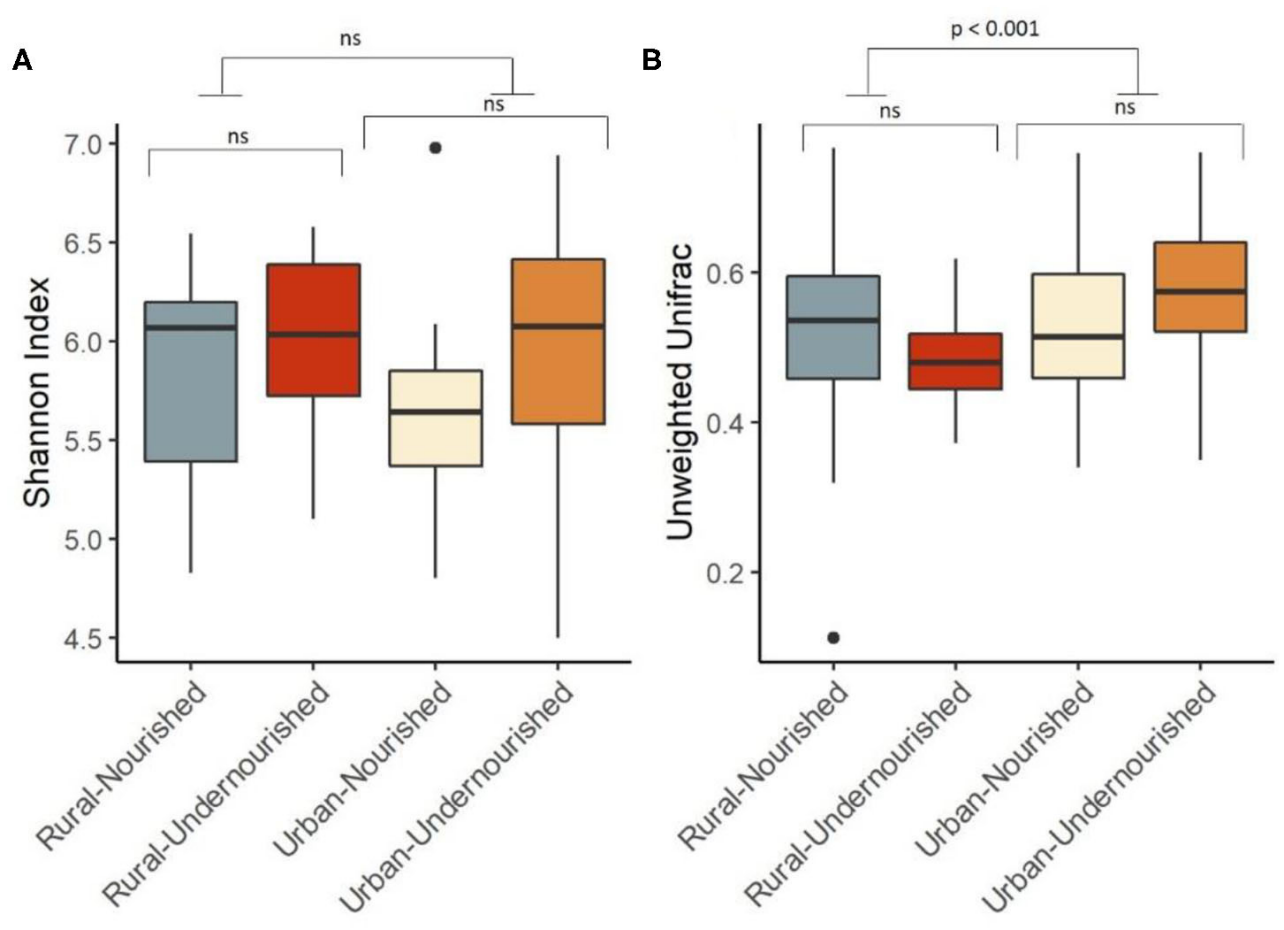
Box and Whisker plot depicting. **(A)** Shannon Diversity (α diversity index) in undernourished and nourished from urban and rural settings; **(B)** Beta Diversity (unweighted Unifrac distances) in undernourished and nourished in urban and rural settings. *p*-values indicate statistical significance from independent *t*-tests comparing nourished and undernourished study groups in both the locations (i.e., rural normal vs. rural undernourished and urban normal vs. urban-undernourished) and independent *t*-test based on location (i.e., rural vs. urban-slum). Box-and-whisker plots show high, low, and median values, with lower and upper edges of each box denoting first and third quartiles, respectively. ns indicates non-significant *p*-values. Black dots represent the outliers.

**Figure 3:**
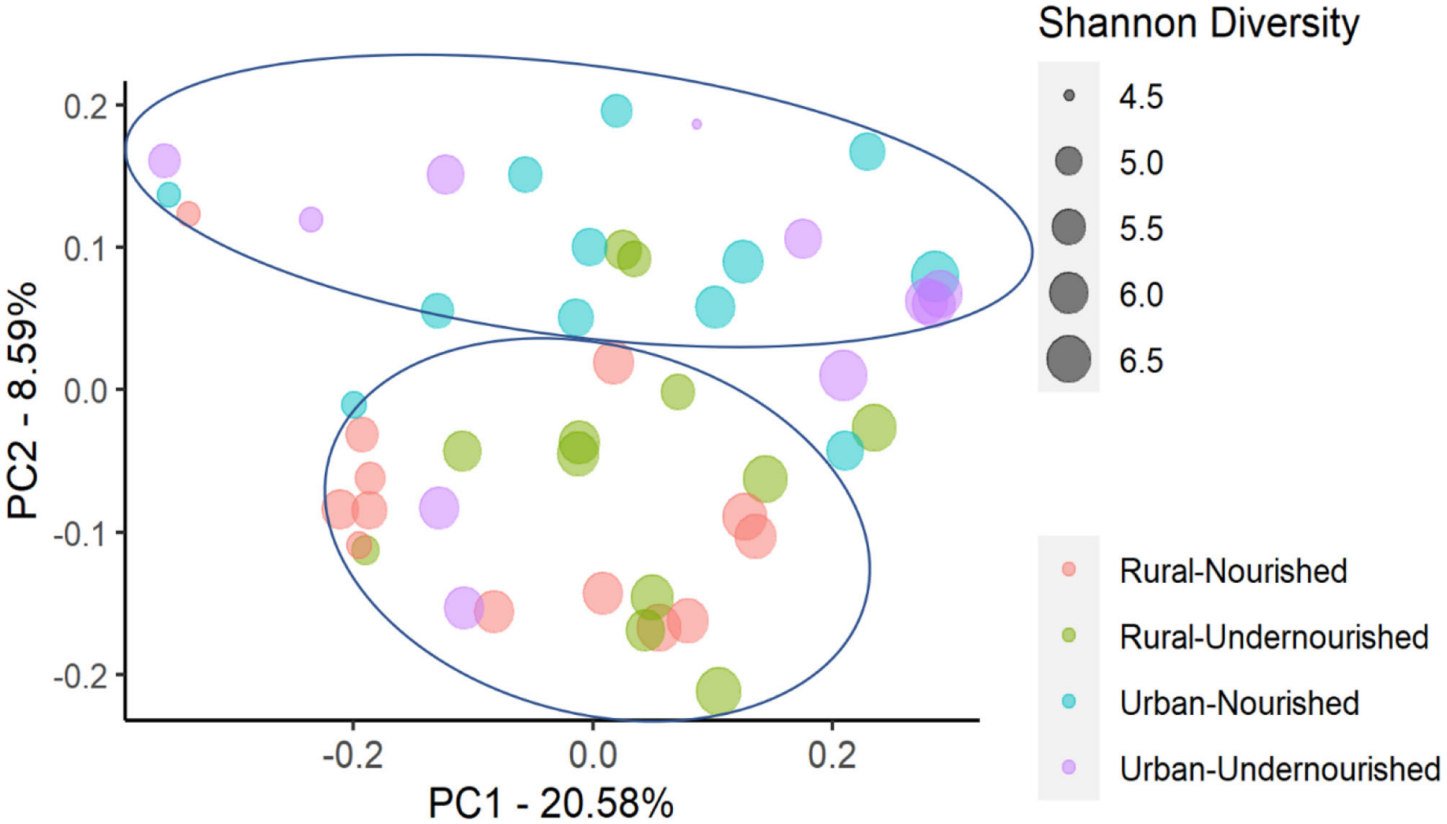
Principal Coordinates Analysis (PCoA) of the similarity across the study groups based on the nutritional status (nourished vs. undernourished) and location (rural vs. urban slum) based on the Unweighted UniFrac distances. Each point corresponds to a sample colored according to the nutritional status and corresponding location and the size of the point corresponds to the Shannon diversity index. Principal Coordinates (PC) axis expresses the percent variance across the samples.

On average, the most dominant phyla were Firmicutes (68%) followed by Bacteroidetes (18%), Proteobacteria (8%), and Actinobacteria (6%). There were no significant differences between the dominant phyla based on nutritional status as shown in Table 1B. However, there was a significant increase in the abundance of Firmicutes and Proteobacteria in the rural and urban slum cohort, respectively, as shown in Figures 1A,B (details in Supplementary Table 1A and Supplementary Figure 1). After taxonomic assignments to the family level, a total of 111 families were found. The independent *t*-tests on the rarefied table resulted in eight statistically significant families based on location which mostly belonged to the phyla Firmicutes and Proteobacteria (Supplementary Table 2). Undernourished adults showed higher alpha diversity (Shannon Index) compared to nourished in both geographical settings but this was not statistically significant (Figure 2A). No significant differences were observed in beta diversity (unweighted UniFrac) between undernourished and nourished in rural or urban slum settings, however, there was a significant difference in the beta diversity based on location (i.e., rural and urban slum) as shown in Figure 2B and Supplementary Figure 2. Specifically, urban slum settings showed significantly higher beta diversity (*p* < 0.001) compared to rural settings. This is mirrored by the Principal Coordinates Analysis (PCoA) plotted based on the unweighted UniFrac distances (Figure 3), demonstrating greater variation within the urban slum cohort and homogeneity in the rural cohort.

### Location Is the Strongest Determinant of the Gut Microbiota Variation Across the Samples

**Figure 4:**
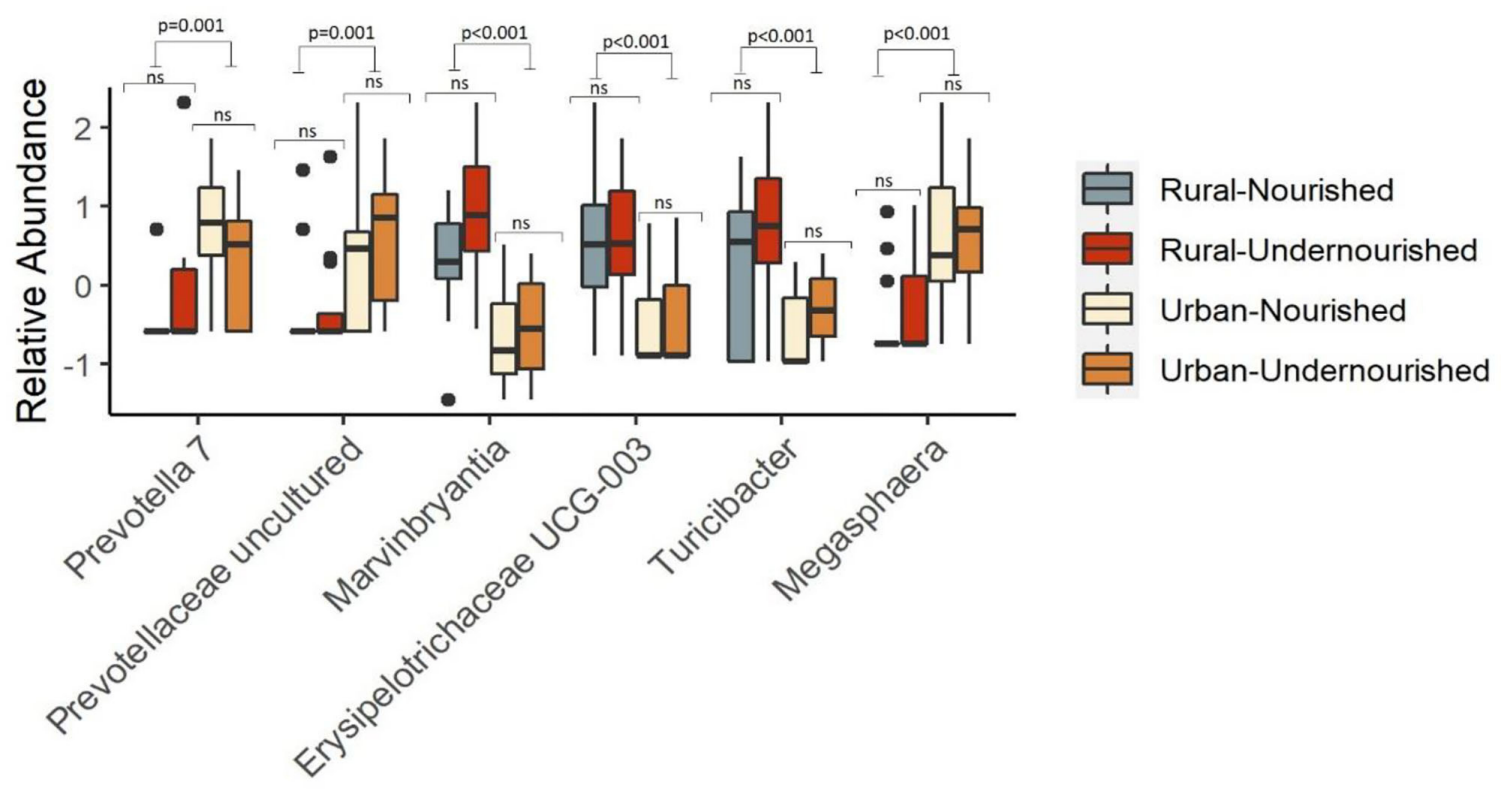
Box and Whisker plot depicting the relative abundance of significant taxa associated with undernourished and nourished from urban slum and rural settings. *p*-values indicate statistical significance from independent *t*-tests comparing nourished and undernourished study groups in both the locations (i.e., rural normal vs. rural undernourished and urban normal vs. urban-undernourished) and independent *t*-test based on location (i.e., rural vs. urban-slum). Box-and-whisker plots show high, low, and median values, with lower and upper edges of each box denoting first and third quartiles, respectively. ns indicates non-significant *p*-values. Black dots represent the outliers.

There were no differentially abundant taxa when tested based on nutritional status. However, taxa significantly associated with the rural settings were *Erysipelotrichaceae UCG-003, Turicibacter*, and *Marvinbryantia*. In the urban slum region, the taxa that were significantly associated belong to the genus *Megasphaera, Prevotella*, and *Prevotellaceae* family as shown in Figure 4. There were no taxa significantly associated with the estimated intakes of carbohydrates, proteins, and fats after correcting for multiple testing at the threshold of FDR < 0.05.

### KEGG Orthologs and Functional Pathways

Based on the 16S rRNA marker gene amplicon data, we compared the functional gene content based on nutritional status and geographical locations, respectively. Although alpha diversity based on the genus level did not show a statistical difference in either of the study groups, reports suggest there may be differences at the functional level (47, 48). To gain a deeper insight into whether microbiota compositional differences may have effects within the subgroups, we computed the abundance of higher functional categories (based on KEGG ortholog abundances).

**Figure 5:**
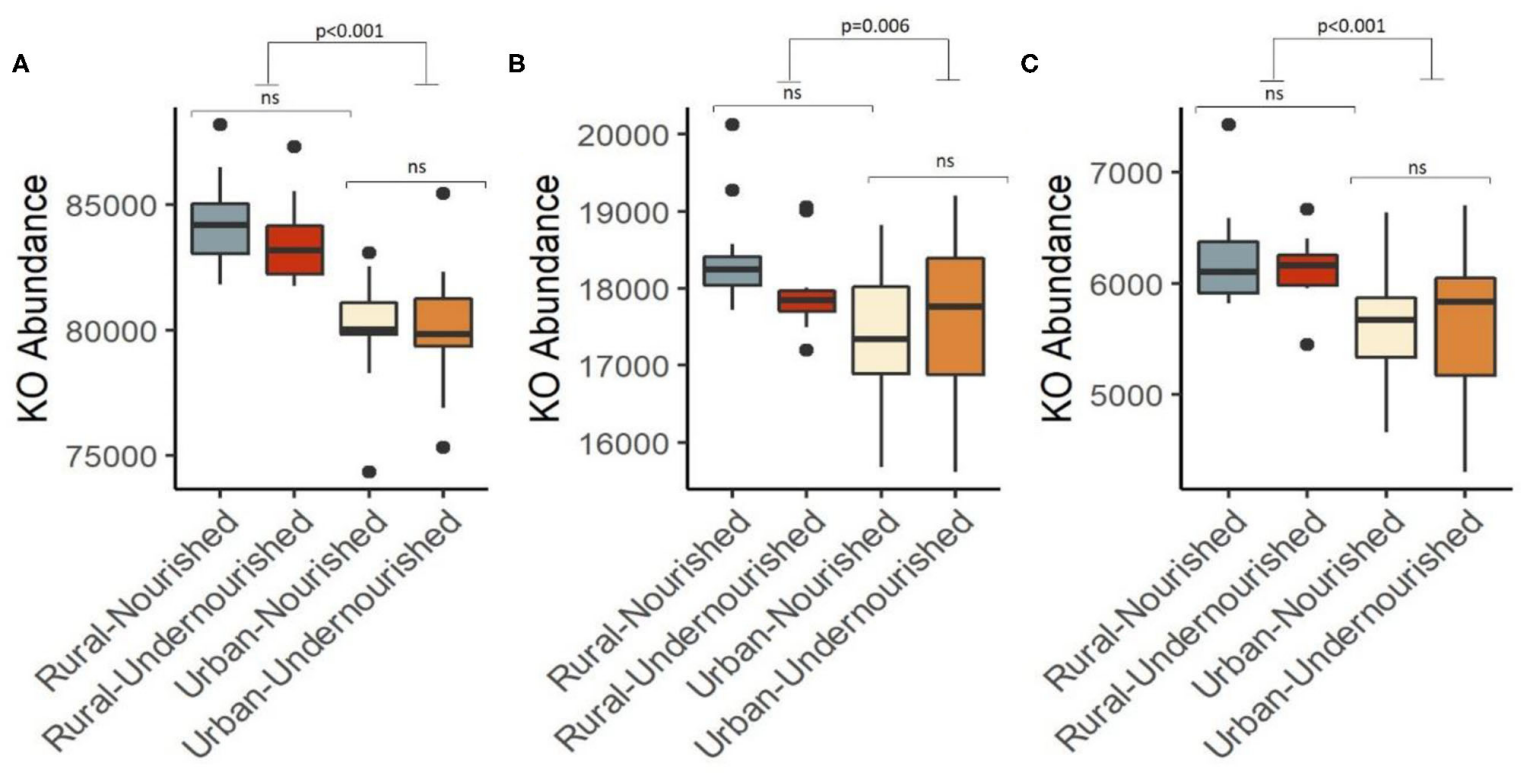
Box and Whisker plot depicting the pathways significantly associated with undernourished and nourished from urban slum and rural settings in **(A)** Carbohydrate metabolism (*p* < 0.001), **(B)** Lipid metabolism (*p* = 0.006), **(C)** Biosynthesis of secondary metabolites (*p* < 0.001). *p*-values indicate statistical significance from independent *t*-tests comparing nourished and undernourished study groups in both the locations (i.e., rural normal vs. rural undernourished and urban normal vs. urban-undernourished) and independent *t*-test based on location (i.e., rural vs. urban-slum). Box-and-whisker plots show high, low, and median values, with lower and upper edges of each box denoting first and third quartiles, respectively. ns indicates non-significant *p*-values. Black dots represent the outliers.

A KEGG ortholog can be a part of many functional pathways, so the functional profiles across the study groups were computed based on the sum of weighted hits of the KEGG orthologs present across the samples in the study groups. Based on nutritional status, there were no significant associations of specific functions, however, pathways for carbohydrate and lipid metabolism, glycan biosynthesis, and nucleotide metabolism along with biosynthesis of other amino acids, metabolism of terpenoids and polyketides, xenobiotic biodegradation, and metabolism were found to be negatively associated with the nourished cohort. Functions for the metabolism of amino-acid and energy, metabolism of cofactors, and vitamins, biosynthesis of secondary metabolites were found to be positively associated with the nourished cohort as shown in Table 2. We then looked at the associations with geographical location and found significant associations for the functional pathways of biosynthesis of other secondary metabolites, carbohydrate metabolism, and lipid metabolism (*p* < 0.05), as shown in Figure 5. Specifically, pathways identified for amino acid, nucleotide, lipid, carbohydrate, and glycan biosynthesis metabolism along with xenobiotics biodegradation, biosynthesis for secondary metabolites, and metabolism of terpenoids and polyketides were negatively associated with the rural settings and functions for energy metabolism, metabolism of cofactors, and vitamins and other amino acids were found to be positively associated with the rural settings as shown in Table 2.

**Table 2:** Correlation between the pathways identified and the study groups based on the nutritional status (normal vs. undernourished) or location (rural vs. urban slum).

**Pathways**	**Nutritional status**		**Location**	
	**(Nourished vs. Undernourished)**		**(Rural vs. Urban slum)**	
	**Coefficient**	***p*-value**	**Coefficient**	***p*-value**
Amino-acid metabolism	0.071	0.633	−0.083	0.576
Biosynthesis of secondary metabolites	0.056	0.707	−0.507^**^	<0.001
Carbohydrate metabolism	−0.113	0.445	−0.727^**^	<0.001
Energy metabolism	0.011	0.943	0.089	0.548
Glycan biosynthesis and metabolism	−0.122	0.409	−0.059	0.692
Lipid metabolism	−0.113	0.445	−0.330^*^	0.022
Metabolism of co-factors and vitamins	0.017	0.911	0.074	0.618
Metabolism of other amino-acids	−0.047	0.753	0.059	0.692
Metabolism of terpenoids and polyketides	−0.047	0.753	−0.254	0.081
Nucleotide metabolism	−0.011	0.943	−0.173	0.239
Xenobiotic biodegradation and metabolism	−0.014	0.927	−0.05	0.737

*Data is expressed as Spearman's coefficient and raw p-values in parentheses*.

* correlation is significant at the level of <0.05;

** *correlation is significant at the level of <0.001. The p-values are significant after correcting for multiple testing at the threshold of ≤0.1*.

## Discussion

In the current study, we looked at the impact of nutritional status and geographical location of residence as possible factors influencing the composition and function of the gut microbiome of adults in Karnataka, India. We find no significant differences in microbial diversities (Shannon and Unweighted UniFrac) between undernourished (BMI < 18.5 kg/m^2^) and nourished individuals (BMI in the range 18.5–25 kg/m^2^) from either rural or urban slum locations even though calorie, carbohydrate, and protein intake was significantly lower in the undernourished group (Table 1A). This may be because, in adults, the core microbiome becomes stable over time (49, 50), which tends to change with the effect of environment and other factors (51, 52). However, beta diversity was found to be significantly different between locations (rural and urban slum settings) and we found several differentially abundant microbial taxa suggesting that location has significant effect in modulating the composition of the microbiome and associated metabolic pathways as reported previously (53). This could be due to substantial differences in food availability, environment, and social practices between the urban slum and rural settings. For example, urban slum settings comprise predominantly of overcrowded and ill-ventilated houses with poor sanitation practices and lower socioeconomic status compared to the rural settings. Furthermore, the people in the urban slum generally have a diet comprising mainly processed foods while the rural population has a higher socioeconomic status with a healthier living environment and a diet comprising fresh traditional staple foods (54, 55). This is reflected in lower average calorie intakes per day in the rural settings compared to the urban slum, however, we observe similar protein, carbohydrates, and fat intakes. Our work supports previous suggestion that extrinsic factors beyond nutritional status appear to be associated with microbial profiles (23–25, 40, 47).

In the current study, a significant increase in Proteobacteria and decrease in Firmicutes in the urban slum settings may indicate associations with the risk of diseases as reported previously (56–58). Also, the significant association of genera such as *Prevotella* and the Prevotellaeceae family in the urban slum settings may reflect the differences in the enzymes responsible for the breakdown of complex indigestible polysaccharides (59). The family Prevotellaeceae and different oligotypes of the genus *Prevotella* have been previously shown to be associated with different dietary patterns (60) and with rural and urban locations (47, 53, 61). In the current study, we also find significant increases in butyrate producing genera such as *Marvinbryantia* and *Turicibacter* associated with the rural location which may indicate a fiber rich diet. The higher-level functional pathways related to carbohydrate, protein and lipid metabolism were also found to be significantly associated with the rural location compared to urban slum location. These differences could be attributed to migration from rural to urban settings which has been previously reported to impair metabolic pathways (62) and gut microbiome composition (63, 64). Although, the gut microbiome has been shown to affect the metabolism of macronutrients with the help of enzymes such as of CAZymes, deaminases, lipases, and others maintaining the homeostasis of metabolic pathways (65), however, the causal factors contributing to this remain speculative. In addition, the positive association of the xenobiotic degradation pathways and the pathways for the metabolism of terpenoids and polyketides with the urban slum population likely reflects the exposure to industrial products as these molecules exist as glycosides in their natural forms and are chemically engineered in the pharmaceutical and nutraceutical industries (66, 67).

Various previous studies have reported no change in diversity metrics but a change in functional profiles that are associated with specific metabolic pathways (68, 69). Our observed variation in beta diversity, differences in specific microbial taxa and functional pathways probably reflect the intake of substantially different dietary resources in rural and urban slum dwellers (e.g., processed foods more commonly available in urban location).

There are substantial strengths to our study. This is the first study that has investigated the role of nutritional status and location of residence on the composition of the gut microbiome in undernourished and nourished adults. The inclusion of undernourished and nourished individuals from two distinct locations within the same region enabled us to gain a deeper understanding of the impact of nutritional status and environment on the composition of the gut microbiome. Also, we have inferred the functioning of the gut microbiome from the 16s rRNA marker-based functional profiling tools. Few studies have compared the outputs of pipelines such as PICRUST, Tax4Fun (70, 71) with shotgun metagenome sample sequencing generating comparable results thus making the process cost-effective against the shotgun metagenomic sequences (47, 72). Although there are many caveats of the functional profile generated from the 16S rRNA marker gene-based microbial profile, the current analysis provides some indication that the gut microbiome although not significantly different in diversity may still alter metabolic capacity by producing secondary metabolites that alters gene expression and associated metabolic pathways. Our study has several limitations. Firstly, the small sample size reduces the power to show significant associations and therefore needs to be replicated on larger sample size. Secondly, dietary data was limited to a single administration of a 24-h food recall diary rather than a standardized Food Frequency Questionnaire (FFQ) restricting analysis of dietary patterns and duration of nutritional status between urban slum and rural populations. Furthermore, the study also lacked detailed information on lifestyle as a specific questionnaire on lifestyle was not administered. The current study also lacked biomarkers for the cross-validation of nutritional status and the composition of the gut microbiome.

In conclusion, the current study suggests that in adults, the composition of the gut microbiome is driven more by the demographic settings of residence than nutritional status.

Contrary to previous studies that have shown significant differences in the microbial diversities of the gut microbiome amongst undernourished children (18, 19), no associations were observed in undernourished adults from either rural or urban settings. Microbiome differences associated with the location of residence and differences in host metabolic pathways may have important consequences on human health influencing metabolism, immunity, development and behavior (73). It may therefore be of value to consider environmental and social factors to address dysbiosis in adults in LMICs. This may enable some health improvement in undernourished individuals. Further studies will be required to understand the interplay between the nutritional status and residual location, socioeconomic factors, and gut microbiome for improved health in adults.

## Data Availability Statement

The datasets presented in this study can be found in online repositories. The names of the repository/repositories and accession number(s) can be found below: BioProject database [Submission ID: SUB8578917, BioProject: PRJNA691318].

## Ethics Statement

The current study involving human participants was reviewed and approved by the Institutional Review Board of Bangalore Baptist Hospital (approval gained on 26.06.2018) and the Health Ministry Screening Committee (HMSC-2018-0548/F1). The current protocol was also approved by the Health Ministry's screening committee (HMSC) through the Indian Council of Medical Research (ICMR), Ministry of Health and Family Welfare. The patients/participants provided their written informed consent to participate in this study.

## Author Contributions

LI, GN, AMV, and AV conceived and designed the study. OM, LI, and AV wrote the manuscript. OM and SA processed and analysed the sequencing data. OM carried out functional profiling. LR and GN collected samples and clinical data. JG and GA reviewed and edited the manuscript. The views expressed are those of the authors and not necessarily those of the NHS, the NIHR or the UK Department of Health. All authors have read, critically reviewed, and approved the final version.

## Conflict of Interest

AMV is a consultant for Zoe Global Ltd., and a member of the scientific advisory board of CPKelco. GA has served as a consultant and an advisory board member for Pfizer and Glaxo SmithKline; he has been a consultant to Amryt Pharmaceuticals and Astra Zeneca.

The remaining authors declare that the research was conducted in the absence of any commercial or financial relationships that could be construed as a potential conflict of interest.

## Supplementary Material

Supplementary Figure 1 Pie Chart representation of the relative abundance of the most dominant bacterial phyla. **(A)** Across all the study groups, **(B)** Across the undernourished and nourished from rural and urban slum settings, and **(C)** Across the location (rural and urban slum).

Supplementary Figure 2 Box and Whisker plot depicting Beta Diversity (unweighted Unifrac distances) based on location. *p*-values indicate independent *t*-tests statistical significance categorizing the study groups based on the location (i.e rural vs urban slum). Box-and-whisker plots show high, low, and median values, with lower and upper edges of each box denoting first and third quartiles, respectively. ns indicates non-significant *p*-values. Black dots represent the outliers.

Supplementary Table 1 Characteristics of the study participants based on the location (rural vs. urban slum). **(A)** Clinical Traits, **(B)** Bacterial phylum abundances expressed as relative abundance. The data is expressed as Mean (Standard Deviation). *p*-values are for independent sample *t*-tests. ^**^*p*-values significant at the level of ≤0.001; ^*^*p*-values significant at the level of <0.05.

Supplementary Table 2 Abundances of taxa at the family level based on location (i.e., rural vs. urban slum). The data is expressed as Mean (Standard Deviation). *p*-values are for independent sample *t*-tests. ^**^*p*-values significant at the level of ≤0.001; ^*^*p*-values significant at the level of <0.05.
