# Supplementary figures and images for: Gut Microbial Profile Is Associated With Residential Settings and Not Nutritional Status in Adults in Karnataka, India
Source: Front Nutr. 2021 Feb 23;8:595756. doi: 10.3389/fnut.2021.595756 (PMC7940358; doi:10.3389/fnut.2021.595756)

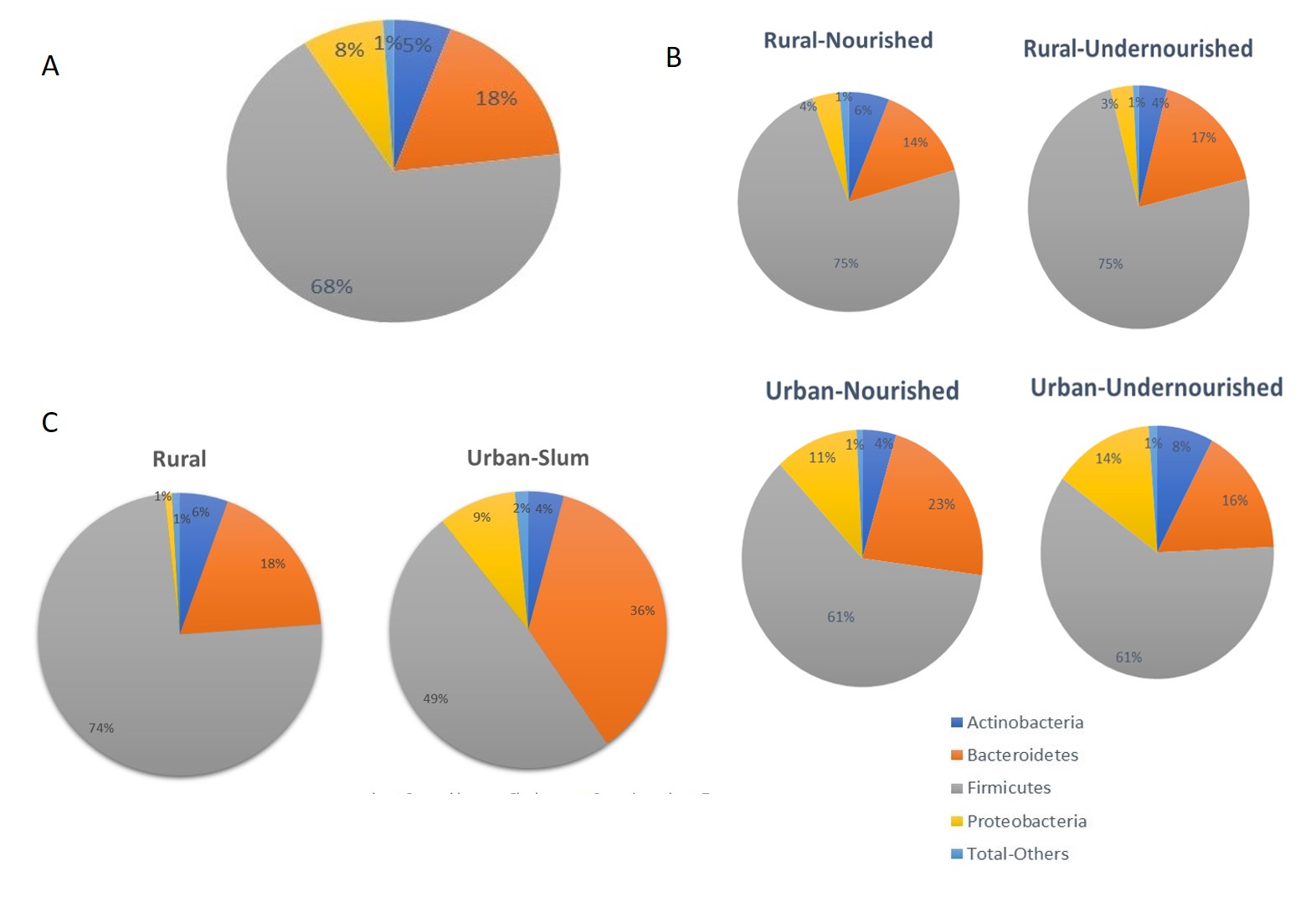

Supplement: Supplementary Figure 1 — Pie Chart representation of the relative abundance of the most dominant bacterial phyla. (A) Across all the study groups, (B) Across the undernourished and nourished from rural and urban slum settings, and (C) Across the location (rural and urban slum). [file Image_1.jpg]

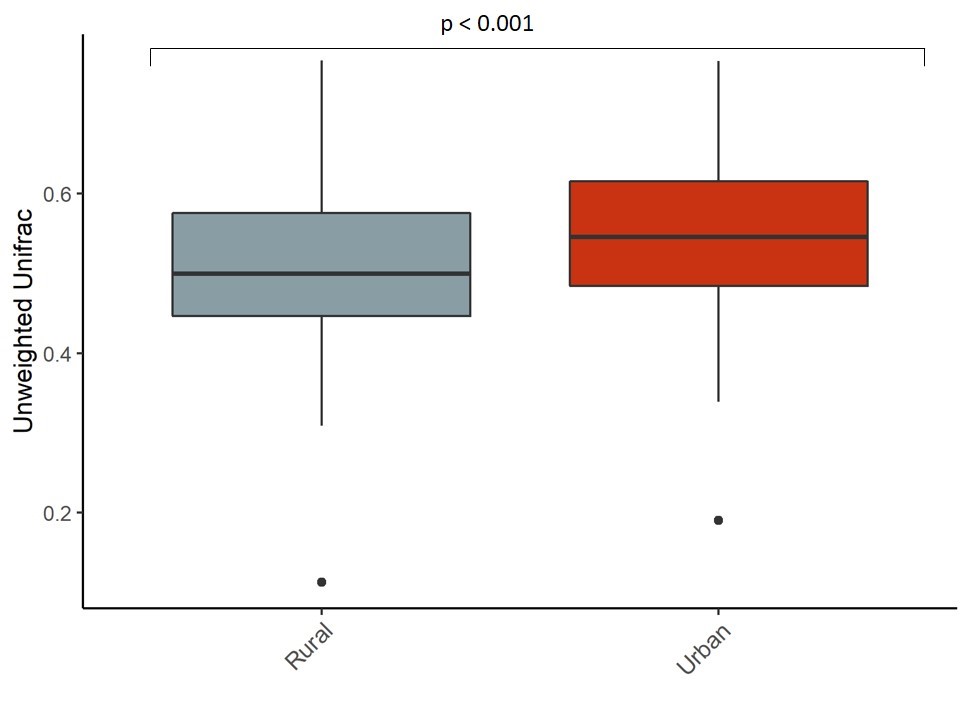

Supplement: Supplementary Figure 2 — Box and Whisker plot depicting Beta Diversity (unweighted Unifrac distances) based on location. p-values indicate independent t-tests statistical significance categorizing the study groups based on the location (i.e rural vs urban slum). Box-and-whisker plots show high, low, and median values, with lower and upper edges of each box denoting first and third quartiles, respectively. ns indicates non-significant p-values. Black dots represent the outliers. [file Image_2.jpg]
